# Socioeconomic differences in use of public, occupational and private health care: A register-linkage study of a working-age population in Finland

**DOI:** 10.1371/journal.pone.0231792

**Published:** 2020-04-16

**Authors:** Jenni Blomgren, Lauri J. Virta

**Affiliations:** 1 Research Unit, The Social Insurance Institution of Finland (Kela), Helsinki, Finland; 2 Research Unit, The Social Insurance Institution of Finland (Kela), Turku, Finland; Charles Sturt University, AUSTRALIA

## Abstract

There is little knowledge on socioeconomic differences in use of health care organized by different care schemes and on exclusive and concurrent use of health care at different schemes in different socioeconomic groups. In Finland, public, occupational and private schemes offer parallel outpatient primary health care services. Each scheme mainly reaches different population groups because of differences in availability, costs and gatekeeping. This study aimed to analyse how the probability of using health care organized by the three schemes differed by socioeconomic status in a working-age population. Individual-level register-based data on use of public, occupational and private outpatient primary health care during 2013 as well as data on sociodemographic covariates were linked for the total population aged 25–64 of the city of Oulu, Finland. Data were analysed with descriptive methods and multinomial logistic regression models. Those in the study population most often used only occupational care or only public care, or did not use any of the studied health care schemes at all. The lower the socioeconomic status, the higher was the probability of not using care or using only public care. The higher the socioeconomic status, the higher was the probability of using occupational care–either only occupational care or occupational care in combination with private care. Education, occupational class and income were all associated with care use also when adjusted for sociodemographic covariates and chronic disease, but income proved to be the strongest predictor of the three. The results reflect the design of the Finnish health care system, with a strong occupational health care scheme for the employed population contributing to inequality in use of health care and potentially to health inequality between socioeconomic groups.

## Introduction

Equity of access to health care implies that health care services are accessed and utilized strictly according to need [1]. Thus, scarce health care resources of societies should be allocated equitably to those most in need, who are often persons in less favourable socioeconomic positions. However, several comparative studies on European and OECD countries have shown that use of health care is strongly associated with socioeconomic status irrespective of need of care: those in a more favourable socioeconomic position are more likely to use doctor services when differences in care needs are adjusted for [2–6]. In these studies, Finland has been among the most unequal countries in terms of doctor visits.

However, only few studies have examined socioeconomic differences in use of health care utilizing data simultaneously on different health care funding schemes. In most countries, the care choice is made between public and private sectors, and the organization of the care system may influence the overall patterns of care use in different socioeconomic groups [7]. Spanish studies using data from 2003 and 2006 have shown that use of public health care was more common among those with low socioeconomic status but use of private care was more common in higher socioeconomic groups [4,8]. Also in Great Britain, use of private care has been found to be more common among those in higher occupation-based socioeconomic groups [8] and among those with higher income [9,10]. However, use of public care was rather equally distributed in Great Britain according to a survey conducted in 2004–2005 [8]. A Swedish study using data from year 2006 found that public general practitioner visits were unrelated to income among working-age men but more prevalent among working-age women in the lowest income quintile; however, private specialist visits were more common among higher income groups among working-age men but unrelated to income among working-age women [11]. In sum, there is a lot of variation in the results from different Western European countries, which is not surprising given their different systems of organizing health care.

Most previous studies on socioeconomic differences in use of health care have included income as the indicator of socioeconomic status [3,4,6,12], but the roles of education and occupational class have only rarely been assessed [4,8]. It has been acknowledged that income-related inequity in use of health care may not always arise from income per se, but may largely be associated with and explained by education or occupational status [12]. Also, different aspects of socioeconomic status may have differential effects on the probability of using health care. Income is directly related to the ability to pay for health services. Education, on the other hand, is associated with knowledge and skills required to be able to navigate in the care system–for example to demand health care, to arrange and keep appointments and to communicate with the care personnel to express care needs [2,7,13]. Depending on the care system, also occupational class may be associated with care use through the availability of different services for different population groups, which is the situation in Finland (see below). It is important to examine various dimensions of socioeconomic status in order to trace the key mechanisms through which inequality in use of health services is produced.

Previous studies have investigated socioeconomic differences in use health care in systems with duplicate (public and private) coverage of care [4,8,11,13]. However, the Finnish system is unique with its triple coverage of outpatient primary health care: besides public and private care, occupational health care constitutes an additional, and nowadays often the most important, primary health care provider for the employed population. In practice, these three rather distinct schemes tend to provide care for different segments of the population and have differential accessibility, price and quality. The Finnish tripartite health care system has been claimed to be a major reason for the high inequity in access to health care in Finland [14–17].

The core of the Finnish care system is *public health care* with universal coverage for all residents. Public outpatient primary health care is organized by municipal health centres and funded by taxes and moderate or no user fees [14,18]. However, gatekeeping may be strong: need for care is strictly screened by a nurse at the point of contacting the health centre. Waiting times for non-urgent appointments may be long. Choice of care provider, such as a specific health centre or a certain physician, is limited. Access to a specialist is granted only through a referral from a general practitioner. Every citizen in need of health care is entitled to public health care, but many prefer to use the other more quickly accessible care schemes, especially occupational health care, if possible.

*Occupational health care* provides services for those in employment. Irrespective of the number of employees, employers are obliged by law to organize, at the minimum, work-related preventive occupational health care for all employees, such as monitoring of working conditions at the workplace and medical examinations. However, depending on the employer, the scope of the services may be even wider. About 80% of employees are also covered by contracts guaranteeing additional curative primary care services that are free of charge for the user at the point of delivery, and most often accessible with very short waiting times [19]. All employees in a certain organization, irrespective of their occupational position, are covered by the same occupational health care contract; however, the exact contents of the service package may vary between employers. Also entrepreneurs may organize occupational health care for themselves. In practice, occupational health care mostly offers the same type of primary health care services that are offered also at the public sector, such as general practitioners’ consultations and curative care. Expenses of occupational health care are mainly covered by the employers and through tax-natured payments by all taxpayers. Funding is partly channelled through the National Health Insurance.

Finally, use of *private health care* is available for all those who are willing and able to pay high user fees in exchange for quick access, free choice of doctor and practically no gatekeeping. Also specialists can be directly accessed at the private sector without referral of a general practitioner. Use of medically necessary private health care is partly subsidized by the state through the National Health Insurance scheme–in 2018, the reimbursement proportion for the patient was, on average, 16% of the costs [20]. Thus, use of private care is largely paid out of pocket and highly correlated with the care users’ income [21].

Previous studies, conducted in settings where the care choice is made between the public and private health care schemes, have examined these schemes separately. There is little if any knowledge on using health care at only one of the available schemes, using several schemes in combination, or not using health care at all, especially in different socioeconomic groups, and including several socioeconomic measures which may reflect different dimensions of socioeconomic status. However, all available schemes should be examined in tandem in order to see the total palette of health care use in different population groups. Especially those who don’t use any health care services can only be observed when all health care schemes are studied at the same time.

Our study aims to fill the above-mentioned gap in knowledge by examining users health of care organized by the three parallel outpatient primary health care schemes in Finland. The aim of this study was to analyse exclusive and concurrent use as well as not using outpatient primary health care on the public, occupational and private schemes among working-age persons in different socioeconomic groups measured by education, occupational class and income. Compared to previous studies that have looked at public and/or private health care schemes, our analyses extend the knowledge by incorporating also a third scheme–the occupational health care scheme. We aimed to quantify the distribution of care users at each health care scheme according to socioeconomic variables, to assess how the socioeconomic variables were associated with use of care when adjusted for age, for chronic disease and for the socioeconomic variables simultaneously, and to see which of the socioeconomic variables was the strongest predictor of health care use. The study aimed to assess the total use of outpatient primary health care; thus we did not restrict the analyses to only doctor visits but included also nurse visits.

## Materials and methods

### Data linkage and study population

Register-based data on health care use and sociodemographic characteristics in year 2013 were collected for the total population of the city of Oulu, situated in Northern Finland. Oulu is the fifth largest city of Finland, with a population of 194 000 inhabitants at the end of 2013. The data set included demographic data and data on income and on chronic disease from the registers of Social Insurance Institution of Finland, data on education and occupational class from the registers of Statistics Finland, data on use of public health care from the City of Oulu, data on use of occupational health care from occupational health care providers, and data on use of reimbursed private health care from the registers of the Social Insurance Institution of Finland. Different data sets were linked through a pseudo-identifier that had been constructed for each individual from the original personal identity numbers prior to granting data access to the researchers. The data were gathered as a joint effort of the Social Insurance Institution of Finland, the Finnish Innovation Fund Sitra, Nordic Healthcare Group, municipality of Oulu and major providers of occupational health care services in Oulu.

The study population consisted of persons who were alive the full year 2013 and were residents of the city of Oulu both at the beginning and at the end of the year. The analyses were restricted to population aged 25–64 measured at the end of 2013, since one of the three studied health care schemes (occupational health care) is practically available only to those in working age. Those aged 65 and over are mainly retired and where thus excluded. Adults under 25 years of age were excluded as their socioeconomic status is often not yet established. Furthermore, students were excluded since there is a separate care scheme for students, for which no individual-level data were available. The final number of persons in the analyses was 92 280 (47 024 men, 45 256 women).

### Measures of outpatient primary health care use

Register data on use of outpatient primary health care services during 2013 (excluding dental care) were collected from the registers of public municipal health care, from providers of occupational health care, as well as from the national reimbursement registers of private health care maintained by the Social Insurance Institution of Finland. The data include visits to and contacts with primary outpatient health care that occurred during year 2013. The measure of health care use was constructed as a combination of use of different schemes, i.e. whether the persons had utilized any of the schemes during the year at least once. Consultations with doctors and nurses were equally taken into account, since task shifting from doctors to nurses is at a comparatively advanced level in Finland [22], and including only doctor care would not give a complete picture of the total use of outpatient primary care. Furthermore, division of work is different at different health care schemes, with particularly the municipal care scheme offering also nurse level care instead of doctor care in straightforward cases. Contacts included actual visits but also house calls and phone and internet consultations. Visits to outpatient clinics of public hospitals, providing specialist consultations that require a referral, were not included. Unfortunately, the number of visits could not be calculated reliably due to inadequate recording of separate visits in occupational care.

### Measures of socioeconomic status

Socioeconomic status was measured with education, occupational class and income. Educational level was categorized into upper tertiary, lower tertiary, secondary and basic level education. According to their occupational class, persons were classified into upper non-manual employees, lower non-manual employees, manual workers, entrepreneurs (including self-employed and owners of companies with salaried employees), unemployed, retired (mainly disability retirees in this age group), and others or unknown [23]. Income was available as the individual’s yearly taxable income in 2013 (earned income, benefit income and income from capital combined, before taxes) and was categorized into five classes based on quintiles of the income distribution of the total study population (1: € 0–15 606, 2: € 15 607–26 777, 3: € 26 778–35 039, 4: € 35 040–47 529, 5: over € 47 529). The same categorization was used for women and men in order to measure equally the purchasing power in both gender groups. Unfortunately, measures of disposable income or income measures taking into account full household income were not available.

Whereas educational level and income can be categorized into rather clear hierarchical classes, occupational class is not a similarly straightforward measure of socioeconomic status: all classes cannot be ordered on a hierarchical scale. Especially, the group of entrepreneurs is not easy to position in the socioeconomic scale as the internal variation of the group is large in terms of, for example, the amount of responsibility related to the size of the company, the number of employees, or the physical heaviness of the work. In sum, when interpreting the results of our analyses, occupational class should not be interpreted as following a hierarchy in the similar manner as education and income. However, it depicts differences between the employed and non-employed groups and also shows differences within the employed population.

### Covariates

The analyses were conducted separately for men and women, given their very different levels of health care use [24] and different patterns of care use by socioeconomic status [11]. Information on age and chronic diseases were used as needs-related factors of health care use. Age was measured at the end of year 2013. Chronic diseases were measured by a proxy variable of entitlements to special reimbursement for medicine expenses. These entitlements, part of the National Health Insurance and granted by the Social Insurance Institution if supported by a relevant doctor’s certificate, guarantee that the patients can obtain the medicines needed for treatment of certain long-term and severe diseases for free or for only a small out-of-pocket share of the cost. These entitlements are often used as a proxy measure for chronic disease [25]. Persons were classified as having chronic diseases if they had entitlements to special reimbursement any time during year 2013.

Table 1 shows the distributions of the study population according to the independent variables. Men were slightly younger and they had slightly less often chronic diseases than women. Women’s educational level (52% tertiary) was higher than men’s (42% tertiary). The proportion of lower non-manual employees was clearly higher among women than among men, while manual workers constituted a clearly larger group among men than among women. Men’s yearly taxable income was higher than women’s. (Table 1)

**Table 1:** Descriptive statistics of the study population.

	MEN		WOMEN	
	N	Distribution (%)	N	Distribution (%)
**Age**				
25–34	12 975	27.6	11 606	25.6
35–44	12 233	26.0	11 018	24.3
45–54	11 412	24.3	11 467	25.3
55–64	10 404	22.1	11 165	24.7
Median age (years)		43		45
**Chronic diseases**				
None	36 417	77.4	33 680	74.4
One	7 267	15.5	8 228	18.2
Two or more	3 340	7.1	3 348	7.4
**Education**				
Upper tertiary	7 840	16.7	8 837	19.5
Lower tertiary	11 676	24.8	14 829	32.8
Secondary	21 234	45.2	17 429	38.5
Basic	6 274	13.3	4 161	9.2
**Occupational class**				
Upper non-manual employee	11 126	23.7	9 825	21.7
Lower non-manual employee	8 545	18.2	18 070	39.9
Manual worker	10 907	23.2	4 717	10.4
Entrepreneur	3 553	7.6	2 130	4.7
Unemployed	6 768	14.4	4 852	10.7
Retired	5 028	10.7	4 718	10.4
Other or unknown	1 097	2.3	944	2.1
**Income group**				
5 (highest)	13 126	27.9	5 330	11.8
4	10 183	21.7	8 274	18.3
3	7 788	16.6	10 667	23.6
2	7 192	15.3	11 263	24.9
1 (lowest)	8 735	18.6	9 722	21.5
Median taxable income (euros)		34 838		27 988
All	47 024	100	45 256	100

Residents of Oulu, Finland, aged 25–64 in 2013.

### Statistical methods

First, we calculated the proportions of persons who had contacts with the three health care schemes during year 2013. Combinations were formed for exclusive and concurrent use of different care schemes according to the socioeconomic variables. Second, multinomial logistic regression models were fitted to examine the adjusted associations of the socioeconomic variables with use of health care in different combinations of schemes. For easier interpretation of the results of the multinomial regression models, the results are shown as average adjusted predictions. These estimates show the predicted probability of being in each group of care use in each socioeconomic group when differences in covariates between the socioeconomic groups have been adjusted for [26,27]. Also, adjusted average marginal effects for care use were calculated from the multinomial regression models to assess the significance of the difference between categories of each socioeconomic variable [26,27]. Akaike's information criterion (AIC) and Bayesian information criterion (BIC) [28,29] from each model were compared in order to assess which of the socioeconomic variables was the strongest predictor of the health care scheme combination. The data were analysed with Stata/SE version 14.2 [30].

### Ethics statement

The study used secondary data retrieved from registers, and no human subjects were contacted to collect the data. According to the General Data Protection Regulation of the EU (GDPR) [31] and the Finnish Data Protection Act [32], processing of personal data is permitted without informed consent for a task carried out in the public interest, such as scientific research. In Finland, an ethical review statement is not required for studies based solely on administrative register data [33]. We followed good scientific practice, data protection guidelines and ethical standards [33] in collecting and analysing the data and in reporting the results. The data were accessed through permissions from the City of Oulu, the Social Insurance Institution of Finland, National Institute for Health and Welfare, and Statistics Finland. The data were fully pseudonymised by the data providers, and the researchers had no access to the personal identifiers of the study subjects. Different data sets were linked through a pseudo-identifier that had been constructed for each individual. There are legal restrictions that prevent from sharing data publicly. Pseudonymised data cannot be openly shared since use of sensitive individual-level health data is strictly regulated by law [32,34] and the data providers have not given permissions for further data sharing.

## Results

### Exclusive and concurrent use of different health care schemes according to socioeconomic status

**Fig 1:**
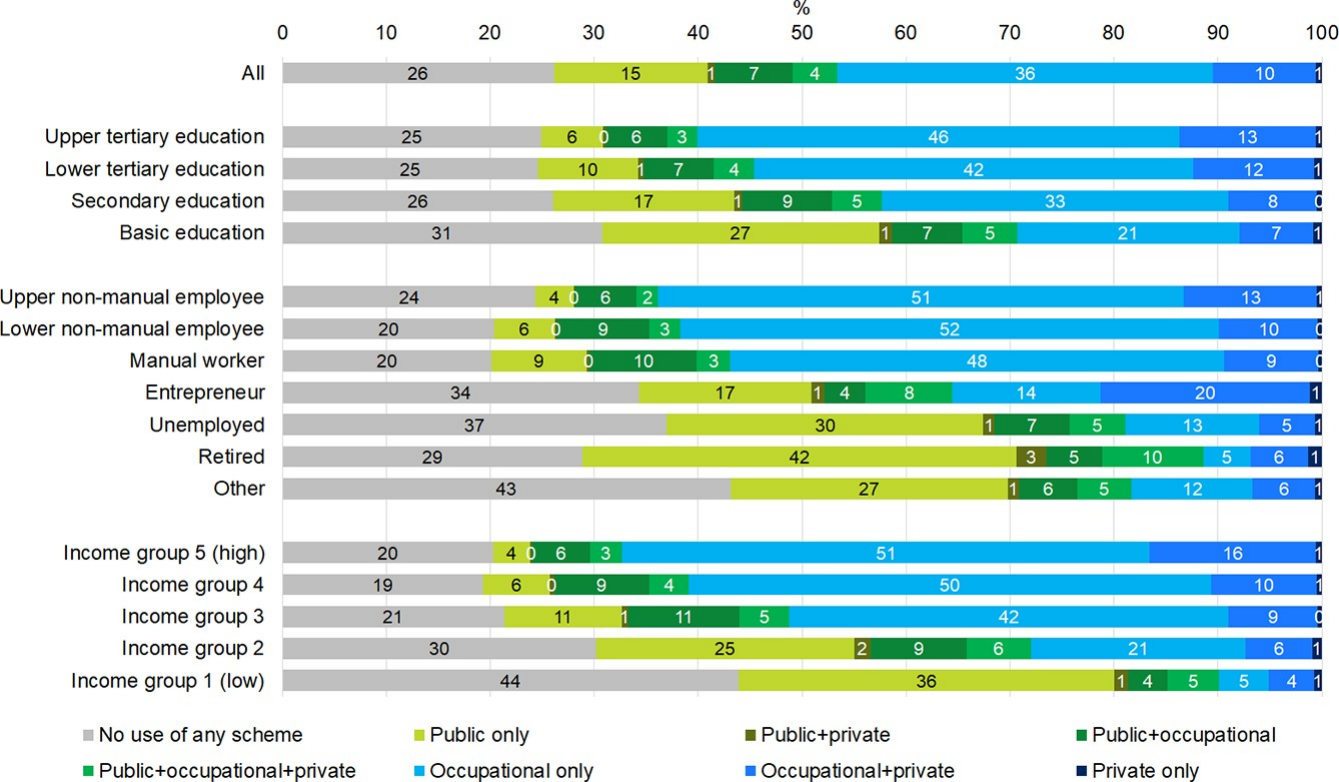
Unadjusted proportions of users of primary outpatient health care schemes during year 2013 among men according to socioeconomic variables. Residents of Oulu, Finland, men aged 25–64 in 2013 (N = 47 024).

**Fig 2:**
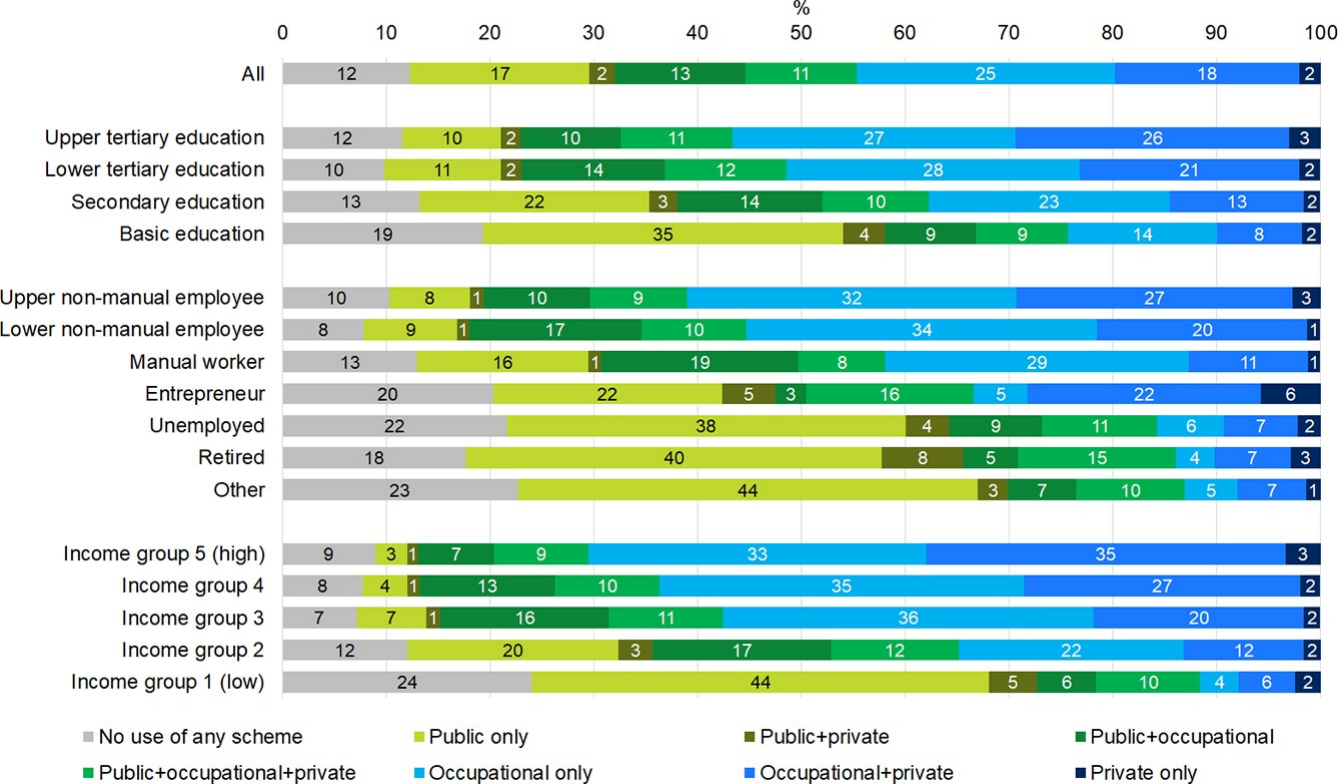
Unadjusted proportions of users of primary outpatient health care schemes during year 2013 among women according to socioeconomic variables. Residents of Oulu, Finland, women aged 25–64 in 2013 (N = 45 256).

Overall patterns of the probability of using health care were quite different by gender (Figs 1 and 2). 26% of men did not use any outpatient primary health care services, 52% used a single scheme and 22% used several schemes. Among women, the same proportions were 12%, 44% and 44%, respectively. The socioeconomic status variables showed a clear pattern as concerns health care use and combinations of schemes among both men and women. Overall, the more adverse the persons’ socioeconomic status was, the more likely they were either to not use any health care at all, or to use only public health care. In contrast, the higher their socioeconomic status, the more likely the persons were to use especially occupational health care–either exclusively or in combination with private health care. However, the differences in the proportions of being in each category of health care users were somewhat smaller by educational level than by occupational class or income.

44% of men belonging to the lowest income group and 43% of men belonging to the occupational class “other” (consisting mainly of persons outside the labour force) did not have any contacts with any primary health care providers during the year, which was around two times more often than among those in the top three income groups or those belonging to the occupational classes with an employment contract. Among women, the proportion of non-users in the least advantaged income and occupational class groups was about one fourth, which was about two to three times the proportion of those in the most advantaged groups.

In the most well-off groups (upper non-manual employees and those in the highest income group), use of occupational health care was remarkably common. In the highest income group, as many as 84% of women and 75% of men used occupational health care during the year (Figs 1 and 2, sections 4–7 of the bars combined). There was some variation in use of occupational health care also between the employed groups, but the main difference by occupational class was–naturally–between the employed vs. the non-employed groups. Using occupational health care contributed to both higher overall use of health care and lower use of public health care among those with the possibility to use occupational health care. However, also in the employed groups there were persons that used only public care (4–9% of employed men (upper and lower non-manual employees and manual workers) and 8–16% of employed women, respectively).

### Adjusted predictions and average marginal effects for being in different user categories of primary health care schemes

**Fig 3:**
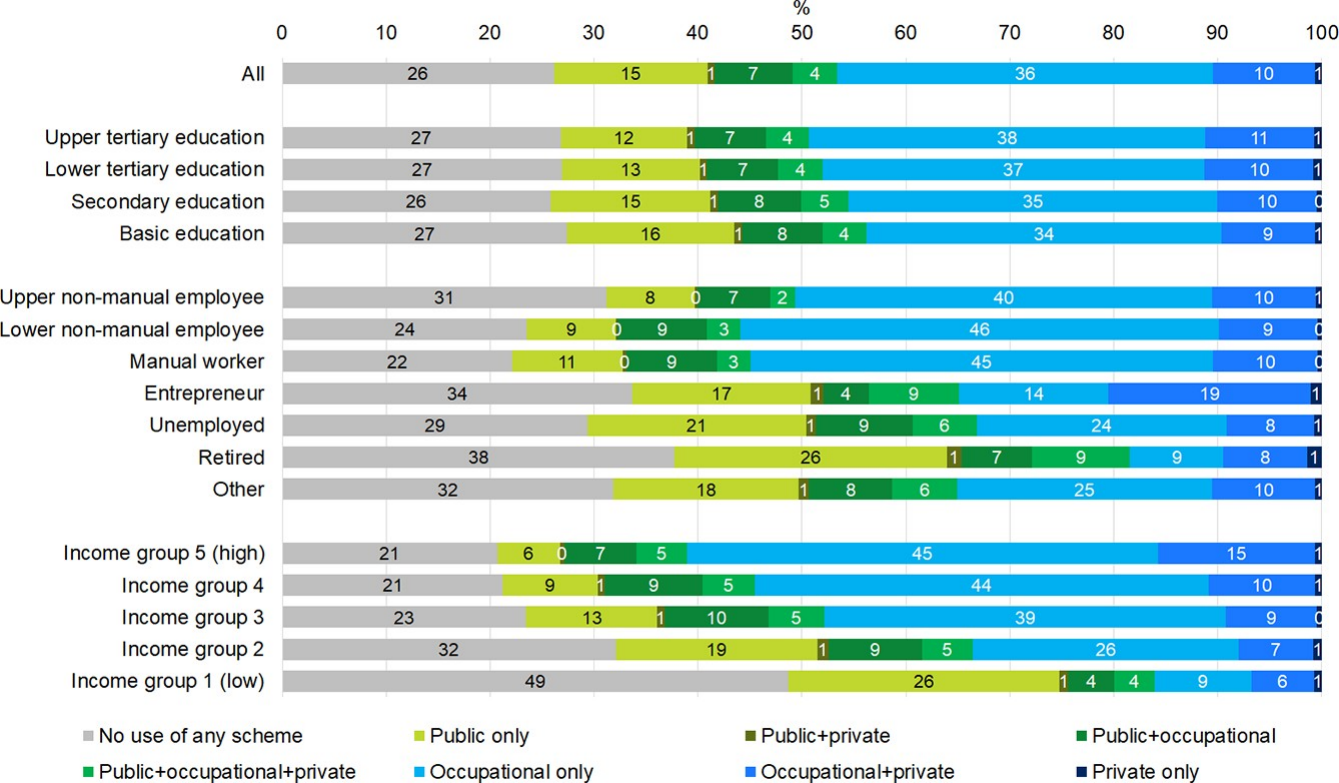
Average adjusted predictions from multinomial logit models of users of primary outpatient health care schemes during year among men 2013 according to socioeconomic variables. Residents of Oulu, Finland, men aged 25–64 in 2013 (N = 47 024).

**Fig 4:**
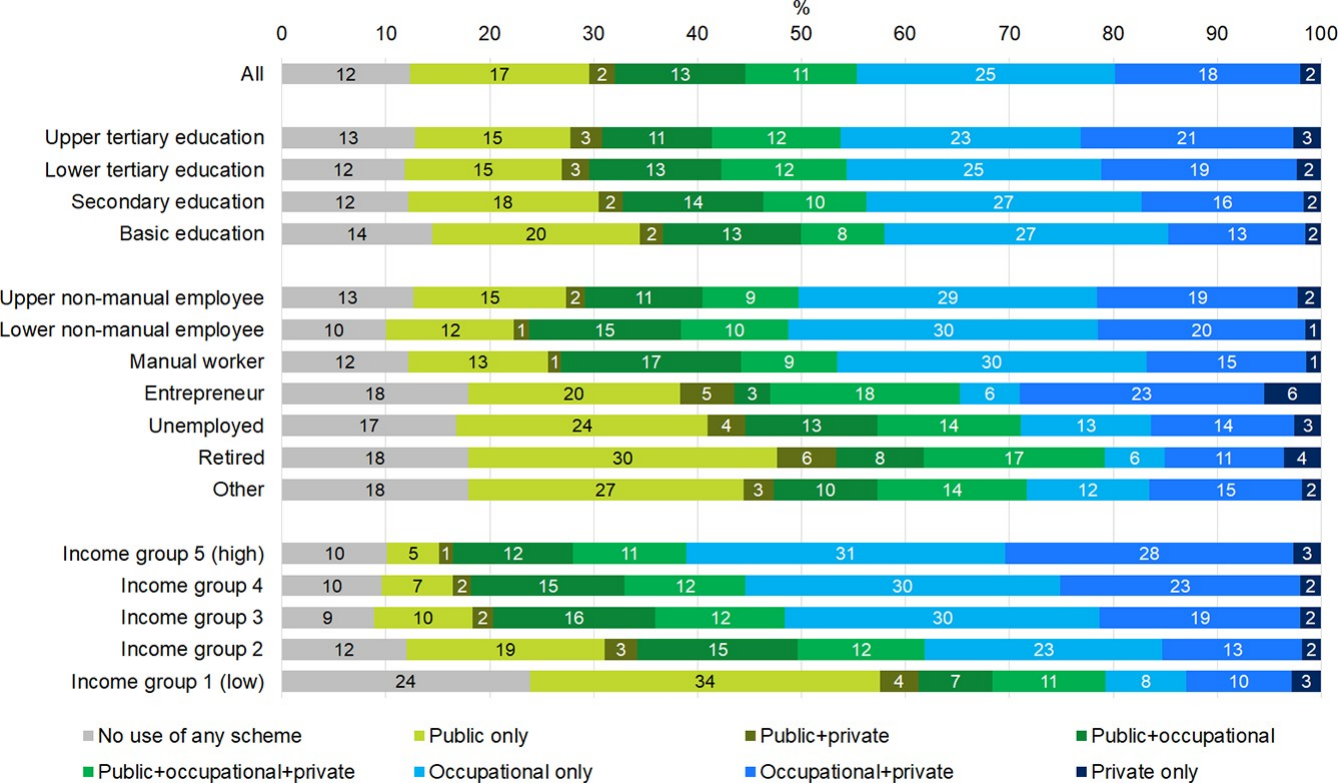
Average adjusted predictions from multinomial logit models of users of primary outpatient health care schemes during year among women 2013 according to socioeconomic variables. Residents of Oulu, Finland, women aged 25–64 in 2013 (N = 45 256).

Figs 3 and 4 show average adjusted predictions for being in the eight care use groups when age, chronic diseases, education, occupational class and income are simultaneously adjusted for in multinomial logistic regression models. When comparing Figs 1 and 2 to Figs 3 and 4, it can be seen that differences by educational groups are nearly explained by other variables, and occupational class differences are somewhat attenuated. However, differences by income are not much affected by adjustments. Stepwise inclusion of variables revealed that attenuation was mostly caused by including the other socioeconomic variables in the models; including age and chronic disease had only a small effect. Even though having chronic disease was associated with a higher probability of health care use, chronic disease was negatively associated with socioeconomic status. Thus, for example the more prevalent use of health care in the higher socioeconomic groups or in the employed vs. non-employed population could not be explained by their higher needs in terms of chronic disease.

Furthermore, average marginal effects were calculated from multinomial logistic regression models adjusted for all covariates in order to estimate the statistical significance of differences in care use between classes of socioeconomic variables. In these models, the smallest groups of care use were combined, and results are shown for five classes (Table 2). The average marginal effects may be interpreted as the adjusted percentage-point differences in the predictions of care use in each class of socioeconomic variables compared to the reference group of each variable. Groups around the middle of the distributions were chosen as references, i.e. those having secondary education, those being manual workers and those belonging to the third income group.

**Table 2:** Adjusted average marginal effects (AME) ^a^ for being in each of the user categories of outpatient primary health care schemes during year 2013 according to socioeconomic variables.

	No use of any health care	Public only	Public combined with occupational and/or private	Occupational only	Occupational combined with private, or private only
	AME (95% CI)	AME (95% CI)	AME (95% CI)	AME (95% CI)	AME (95% CI)
**MEN**					
**Education**					
Upper tertiary	1.0 (-0.4–2.3)	**-3.2** (-4.3– -2.1)	**-1.5** (-2.6– -0.4)	**2.6** (1.2–4.0)	**1.1** (0.2–2.1)
Lower tertiary	**1.1** (0.0–2.1)	**-2.0** (-2.8– -1,2)	**-1.5** (-2.3– -0.7)	**1.3** (0.2–2.4)	**1.2** (0.4–2.0)
Secondary	ref.	ref.	ref.	ref.	ref.
Basic	**1.5** (0.3–2.8)	0.8 (0.0–1.6)	-0.7 (-1.6–0.3)	-1.3 (-2.7–0.1)	-0.3 (-1.3–0.6)
**Occupational class**					
Upper non-manual	**9.1** (7.7–10.6)	**-2.2** (-3.2– -1.1)	**-2.6** (-3.7– -1.6)	**-4.5** (-6.0– -2.9)	0.2 (-0.9–1.2)
Lower non-manual	**1.4** (0.2–2.7)	**-2.1** (-3.0– -1.2)	-0.3 (-1.2–0.7)	**1.5** (0.1–2.9)	-0.5 (-1.4–0.4)
Manual worker	ref.	ref.	ref.	ref.	ref.
Entrepreneur	**11.6** (9.9–13.4)	**6.6** (5.2–8.0)	**1.9** (0.6–3.3)	**-30.2** (-31.7– -28.7)	**10.1** (8.6–11.5)
Unemployed	**7.4** (6.0–8.9)	**10.5** (9.4–11.6)	**3.8** (2.6–5.1)	**-20.6** (-22.2– -18.9)	**-1.2** (-2.3– -0.1)
Retired	**15.0** (13.0–17.0)	**15.3** (13.8–16.9)	**6.2** (4.7–7.8)	**-35.8** (-37.2– -34.3)	-0.8 (-2.1–0.4)
Other or unknown	**9.9** (7.0–12.7)	**7.4** (5.3–9.5)	**2.6** (0.1–5.2)	**-20.0** (-23.6– -16.4)	0.1 (-2.3–2.5)
**Income group**					
5 (highest)	**-2.7** (-4.0– -1.4)	**-6.5** (-7.5– -5.6)	**-4.0** (-5.1– -2.9)	**6.6** (5.2–8.1)	**6.6** (5.6–7.7)
4	**-2.2** (-3.5– -1.0)	**-3.6** (-4.5– -2.6)	-0.9 (-1.9–0.2)	**5.0** (3.7–6.4)	**1.7** (0.8–2.5)
3	ref.	ref.	ref.	ref.	ref.
2	**8.6** (7.2–10.1)	**6.8** (5.6–7.9)	**-1.1** (-2.3–0.0)	**-13.1** (-14.6– -11.6)	**-1.2** (-2.1– -0.3)
1 (lowest)	**25.0** (23.4–26.7)	**13.3** (12.0–14.6)	**-6.5** (-7.6– -5.4)	**-29.4** (-30.7– -28.0)	**-2.4** (-3.3– -1.4)
**WOMEN**					
**Education**					
Upper tertiary	0.7 (-0.4–1.8)	**-3.3** (-4.4– -2.2)	0.3 (-1.2–1.8)	**-3.4** (-4.8–2.0)	**5.8** (4.4–7.1)
Lower tertiary	-0.3 (-1.1–0.4)	**-3.2** (-4.0– -2.4)	**1.7** (0.7–2.7)	**-2.0** (-3.0– -1.0)	**3.8** (2.9–4.7)
Secondary	ref.	ref.	ref.	ref.	ref.
Basic	**2.4** (1.3–3.4)	**1.8** (0.7–2.9)	**-2.6** (-4.1– -1.1)	0.9 (-1.0–2.7)	**-2.4** (-3.8– -1.0)
**Occupational class**					
Upper non-manual	0.5 (-0.8–1.9)	**1.4** (0.1–2.8)	**-6.0** (-7.8– -4.2)	-1.0 (-2.9–1.0)	**5.0** (3.3–6.7)
Lower non-manual	**-2.2** (-3.2– -1.2)	**-1.2** (-2.2– -0.2)	-1.5 (-3.0–0.0)	0.0 (-1.5–1.5)	**4.9** (3.5–6.3)
Manual worker	ref.	ref.	ref.	ref.	ref.
Entrepreneur	**5.6** (3.8–7.4)	**6.9** (5.1–8.6)	-0.8 (-3.1–1.6)	**-24.0** (-25.7– -22.3)	**12.3** (10.2–14.5)
Unemployed	**4.6** (3.2–6.0)	**10.8** (9.5–12.2)	**2.0** (0.0–4.0)	**-17.3** (—19.2– -15.5)	-0.1 (-1.9–1.7)
Retired	**5.1** (3.5–6.7)	**15.5** (13.8–17.2)	**5.6** (3.5–7.8)	**-24.2** (-25.8– -22.6)	**-2.1** (-3.8– -0.4)
Other or unknown	**5.9** (3.3–8.4)	**13.2** (10.7–15.8)	-1.0 (-4.5–2.6)	**-18.0** (-21.3– -14.7)	-0.2 (-3.5–3.2)
**Income group**					
5 (highest)	**1.4** (0.2–2.5)	**-4.3** (-5.3– -3.3)	**-6.4** (-8.1– -4.7)	0.3 (-1.4–1.9)	**9.1** (7.4–10.7)
4	0.8 (-0.1–1.7)	**-2.6** (-3.5– -1.7)	**-2.1** (-3.5– -0.8)	0.0 (-1.3–1.2)	**3.9** (2.7–5.1)
3	ref.	ref.	ref.	ref.	ref.
2	**3.2** (2.3–4.1)	**9.6** (8.7–10.6)	0.6 (-0.7–1.8)	**-7.3** (-8.5– -6.2)	**-6.1** (-7.1– -5.0)
1 (lowest)	**14.8** (13.5–16.1)	**23.8** (22.4–25.1)	**-8.1** (-9.6– -6.7)	**-22.6** (-23.7– -21.4)	**-7.9** (-9.2– -6.6)

Residents of Oulu, Finland, aged 25–64 in 2013.

^a^ AME = average marginal effects from multinomial logit models = %-points difference in the probability of belonging to each category compared to the reference group. Adjusted for age, chronic disease, education, occupational class and income. Bold font = statistically significant difference (p<0.05) compared to the reference group.

In most cases, the adjusted differences between classes of each socioeconomic variable remained statistically significant. The adjusted differences between educational groups were rather small but nevertheless mostly statistically significant and roughly followed a consistent gradient. Differences across occupational classes were large, with the upper and lower non-manual employees and manual workers roughly constituting one group from which the rest of the categories (those not employed) differed. Largest differences were seen in not using care, using only occupational care and in using only public care. Both among men and women, groups without employment contracts were–naturally–less likely to use occupational health care but more likely to use only public health care compared to manual workers and to non-manual employees.

The adjusted differences were most explicit across income groups. Also here, differences were especially pronounced in not using care or using only public or only occupational care–as well as using occupational care combined with private care. For example, compared to men in the middle income group, men with lowest income had a 25 percentage points higher probability of not using any primary health care, a 13.3 percentage points higher probability of using only public care and a 29.4 percentage points lower probability of using only occupational care. When comparing income groups 1 and 5, the differences were naturally even more striking.

Overall, the likelihood of opting out of the public system (using only occupational care, private care or these two in combination, but not using any public health care) was more likely among those with higher education, among those employed (non-manual employees and manual workers), and among those having higher income. In contrast, groups characterized by low education, being outside employment and having low income were more likely to resort to only public care–or, even more strikingly, they often did not use any health care at all.

Comparison of Akaike’s information criterion and Bayesian information criterion values from models including each socioeconomic variable at a time confirmed that income was the strongest and education the poorest predictor of care use of the three socioeconomic variables (Table 3). However, in terms of these criteria, the best model for both men and women was the one including all socioeconomic variables.

**Table 3:** Akaike's information criterion (AIC) and Bayesian information criterion (BIC) values for different sets of models including the socioeconomic variables.

	AIC	BIC
MEN		
Age, chronic disease and education	147 706	148 258
Age, chronic disease and occupational class	137 769	138 505
Age, chronic disease and income	137 391	138 004
Age, chronic disease, education, occupational class and income	133 086	134 251
WOMEN		
Age, chronic disease and education	163 888	164 437
Age, chronic disease and occupational class	155 343	156 075
Age, chronic disease and income	154 147	154 757
Age, chronic disease, education, occupational class and income	149 997	151 157

## Discussion

### Summary of the results

This study reported novel information about the probability of using outpatient primary health care by socioeconomic groups when all relevant health care schemes in one country were assessed simultaneously. Also, the study highlighted results related to the rather unique Finnish occupational health care scheme. Among both men and women, all socioeconomic status variables were remarkably strongly associated with health care use, income being the strongest and education the weakest predictor of the three indicators. In general, the lower the socioeconomic status measured by any of the three indicators, the higher was the probability of using no health care or using only public health care. The higher the socioeconomic status, the higher was the probability of using occupational health care. Using only private care was rare–private care was more often used as a complementary scheme for occupational health care especially in higher socioeconomic groups. The most well-off groups were the most likely to have contacts with at least one of the health care schemes during the year, and most often the scheme they used was occupational health care.

### Results of the study in the context of previous literature

Our study is among the first to quantify socioeconomic differences in use of health care at different schemes simultaneously and to our knowledge the first to incorporate data on a third health care scheme–occupational health care–in the same analyses with data on public and private health care. The results are in accordance with previous studies examining socioeconomic differences in use of health care that have studied individual care schemes separately [2–6,8,10,11]. However, in this study, the role of occupational health care stood out, and it deserves a special assessment in the Finnish context, as overall differences in service use are largely associated with utilizing this scheme. The Finnish tripartite health care system is unique, with three parallel schemes providing outpatient primary health care services. In contrast to many other countries, the role of private health care in the working-age population is small also in the highest socioeconomic groups. Because of the design of the health care system in Finland, occupational health care is usually the primary choice for those in better socioeconomic positions–sometimes complemented with some services acquired from the private scheme.

Our results that show strong socioeconomic gradients when need factors are adjusted for may reflect both overuse of health care in higher socioeconomic groups and underuse of health care in lower socioeconomic groups. It is plausible that both mechanisms are at work. Why was use of health care so common among the well-off groups, even though it is well known that they are healthier than the less well-off groups [35] and thus should not need care as often? First, as especially occupational health care is very quickly available and free for the user at the point of delivery, there are low barriers to use it even for treatment of minor health problems. Second, socioeconomic status is related to the interests and ability to care for one’s health; thus those having a higher socioeconomic status may be more capable to navigate in the system and make and keep appointments [2]. This effect is further intensified by the fact that the occupational health care system is very accessible and free of charge. On the other hand, it has to be noted that not every employed person uses occupational health care. Some may still prefer to use public services because of, for example, a conveniently situated health centre, or a long-term contact with the public health care for example in long-term treatment of a chronic disease. Also in our data, some proportion of the employed population used only public care.

Another factor explaining high prevalence of health care use in the employed groups is that in case of sickness absence, a physician’s certificate is normally required from employed persons after a self-certified period of one to three days [36]. Thus, all visits by the employed groups may not be due to true need of care but due to the requirement of a medical certificate from the fourth day onwards. Also related to the employment contract, employers may organize mandatory physical examinations related to age or to new recruitments, which again increases the employees’ probability of health care visits.

In contrast, those in lowest socioeconomic groups tended to use least health care even though they have on average more care needs compared to those in higher socioeconomic groups [35]. Those with only basic education, with low income and not having an employment contract were more likely to not have any contacts with primary health care. One of the most striking results was that almost one half of men in poorest socioeconomic positions did not have any contacts with primary health care during the year under study. This may partly be due to not easily accessing the necessary services, not using services for minor symptoms when care is not free of charge, not having a possibility to care for one’s health as much as those in higher positions, or not knowing how to navigate in the care system [2]. Also, for those with a less favourable socioeconomic status, out-of-pocket payments may create a financial barrier for health care use [37]. Small out-of-pocket payments (the fee was 13.8 euros per visit in Oulu in 2013) are usually charged for using the universal public health care services in Finland. The large socio-economic differences in non-use of primary health care may also be partly understood through the fact that the well-off employed groups can utilize occupational health care–a scheme that is in many ways more attractive than the two other schemes. Also those in disadvantaged positions would probably use health care more often if the public scheme would be as easily accessible as occupational health care.

The role of the three main aspects of socioeconomic status (education, occupational class and income) has not been simultaneously assessed in previous studies. Previous studies have most commonly used income as the socioeconomic indicator [2–6], but also studies including education [4,7] or occupational status [4,8] have found socioeconomic inequities in the same direction as when using an income-based measure. Our results showed that when all of these three indicators were taken into account simultaneously, income proved to remain the strongest and the most consistent predictor of overall use of health care and of the health care schemes that were used. The associations related to education were almost totally mediated by the other socioeconomic variables. However, inclusion of all three variables is warranted in studies on inequity of health care since educational level, occupational class and income indicate different aspects of socioeconomic status: knowledge and health literacy, availability of different types of services, and ability to pay for these services [2,7,13].

### Aspects of equity and funding of health care

The Finnish social and health security system is based on ideas of universality and equity [16]. Thus, if the so called equity principle was followed, health services should be distributed according to need and not according to socioeconomic status [3,5]. Nevertheless, as we showed in this study, in the current system, use of health care in the working-age population is strongly unequal, mostly because persons in the most disadvantaged positions do not use the services at all or resort to the more scarcely available public care, while the most well off groups may utilize the free and easily available occupational health care, potentially complemented by private care. Opting out of the public scheme among the well of groups may be problematic for the sustainability of the system, since this corrodes these groups’ willingness to pay taxes necessary for funding the system [9].

Some have claimed that as long as the publicly funded health care is equitably distributed according to need [4, 8], an unequal total distribution of use of health care is not so problematic. In the Finnish context, there is no straightforward answer to the question whether occupational health care should be treated as a scheme with separate funding, or whether it should at least partly be considered as part of the public scheme. Occupational health care is funded largely by employers but also from the National Health Insurance through tax-like payments. The opponents of this scheme claim that these resources should be reallocated to the public scheme to benefit everyone. From this point of view, occupational health care represents inequitable allocation of common resources to those most well off in the society. Others claim that since occupational health care is largely funded by the employers, these resources are not directly transferable to fund the public system. This is not an easy puzzle, since cutting down National Health Insurance based funding from occupational health care could diminish or end also the employers’ own funding for the scheme. Thus, discontinuing this scheme could put clearly more pressure to the public health care scheme in organizing health care services for the employed population.

A major reform is being planned for the organization of social and health care services in Finland [38]. According to the plans, instead of the current very scattered public system based on 310 municipalities, the responsibility of organizing primary health care services will lie on larger units, 18 new counties. In the new system, the aim is to improve availability of primary health care services and create more equal opportunities for different population groups to get the services they need. However, according to current knowledge, the occupational health care scheme–jointly funded by employers, employees and National Health Insurance through tax-like payments–will most likely not be reorganized in the reform. Furthermore, additional private health care services will always be accessible to those who are able to pay for them. Thus, despite good intentions, it is unlikely that the planned health care reform will greatly decrease socioeconomic differences in access to health care in Finland. However, this reform, like health care reforms in other countries that have systems based on public care, should strive to enhance the tax-payers’ trust in the public health care system and in its ability to provide necessary health care services for all population groups. This would help increase equality in use of health care and prevent the health care system from polarizing into strong public and private schemes with different accessibility and quality.

### Methodological considerations

We were able to utilize wide-ranging individual-level register-based data on use of care at three primary health care schemes. The data were retrieved from administrative registers that are highly reliable sources, with no non-response or self-report bias and very little missing data. In particular, register data are more reliable than self-reported information for observing use of health care at different schemes since due to the mixed system and recall bias, it is not always easy for the individuals to remember or know which scheme they have actually used. There are no comprehensive registers covering the total health care use of the Finnish population, let alone registers on use of occupational health care, which underlines the uniqueness of the data set compiled for the city of Oulu. Furthermore, we were able to observe health care use over an entire year, which removes the effects of seasonal variation in care use due to, for example, flu epidemics.

However, we could unfortunately not take into account the number of yearly visits due to inconsistencies in recording separate visits in the data on occupational health care. Some previous studies have examined both the annual probability of doctor visits and the annual number of visits [3,6]. These studies have found that inequality in health care use tends to be even more pronounced when examining the number of visits compared to examining only the probability of visits. Thus, it is likely that if the number of visits could have been measured, the observed differences in health care use between socioeconomic groups would have been even larger than those observed in this study.

Due to the observational nature of the data, causal effects could not be established. Confounding by health status or by other unmeasured factors may at least partly explain some of the observed associations. A limitation of the study is the lack of information on needs-related factors. We could use the number of entitlements to reimbursements of medicine expenses as a proxy for chronic disease, but we could not adjust for other health-related factors or for health behaviour. Direct measures of acute disease were not available in these register data but they would be useful in order to achieve better adjustment of need of health care in different socioeconomic groups. On the other hand, use of health care–our dependent variable–is per se an indirect measure of acute disease even though all visits are not due to urgent causes. Thus, it would also be problematic to build a register-based measure of acute disease that is not inherently built in our dependent variable.

Furthermore, we had no information on private health insurance that most probably is associated with use of private services (about one fifth of Finnish adults have private health insurance [39]). Also our income measure has some restrictions. Income was measured as personal income since we had no access to data on equivalent income that takes into account the total household disposable income and household structure. Thus, assuming that income is shared within the household and there are benefits of shared consumption, our income measure may not always reflect the true consumption potential of the individuals. On the other hand, also the other socioeconomic variables in our study were measured at the personal level, not at the household level. An individual’s personal income is an indicator of his/her own socioeconomic status even though the income quintiles are likely to be internally heterogeneous in terms of the true consumption potential. It is plausible that if income quintiles were based on equivalent income, at least the differences between the income quintiles in privately paid health care could potentially be even larger.

As the data were collected from only one city, the results may not be totally generalizable to the total Finnish working-age population. However, as Oulu is the fifth largest city of Finland, it is reasonable to assume that the findings hold at least at the general level for the country in total.

### Future research

This study assessed only the probability of outpatient primary health care consultations during one calendar year. In future studies, a longer follow-up time and including the number of visits at each scheme would perhaps reveal more nuanced patterns of health care use and would, for example, enable tracing population groups that do not use health care at all during a longer time frame. Also, in order to trace the causal effects of socioeconomic status on care use, it would be important to assess the effects of changes in socioeconomic position on changes in care use. Tackling these questions requires longitudinal data over several years. If the laws concerning the planned social and health care reform in Finland pass, this type of study should be replicated after the reform to monitor to which extent a more equal health care system has actually been reached.

### Conclusion

This study showed the importance of simultaneously incorporating all relevant health care schemes in analyses of health care use by socioeconomic status. Also, the study highlighted the implications of the rather unique Finnish occupational health care scheme for overall health care use. We found large socioeconomic differences in use of care at different health care schemes. The results reflect the design of the current Finnish health care system that contributes to inequality in accessibility of health care services. Those who are employed and have sufficient means can utilize any of the three available schemes: public health care, occupational health care and private health care. However, those with no employment and low means are largely dependent on public health care provided by municipal health centres and strikingly often they use no care at all.

In sum, inequity in use of health care in the Finnish context largely arises, on the one hand, from non-use of care in lower socioeconomic groups or their resorting to the often scarcely available public care, and on the other hand, from the ability of the employed and well-off persons to use the freely available occupational health care, potentially complemented with private care. The observed unequal distribution of health care is problematic from the point of view of equity and allocation of scarce resources according to need. Especially, the results showed that easy access to occupational health care for the employed population is one of the drivers of inequality in use of health care. A system with an unequal accessibility of care may also contribute to socioeconomic differences in health. Enhancing timely access to health care may be crucial in order to tackle health problems in the working-age population, especially among those in disadvantaged socioeconomic positions.
